# Supplementary material for: Atherosclerotic-Derived Endothelial Cell Response Conducted by Titanium Oxide Nanotubes
Source: Materials (Basel). 2023 Jan 13;16(2):794. doi: 10.3390/ma16020794 (PMC9865858; doi:10.3390/ma16020794)
Supplement: Supplementary file 1 [file materials-16-00794-s001.zip › materials-2133283-supplementary.pdf]

Atherosclerotic-Derived Endothelial Cell Response Conducted by Titanium Oxide Nanotubes

Ernesto Beltrán-Partida[1], Benjamín Valdez Salas*[1], Martha García-López Portillo[1], Claudia Gutierrez-Perez[1], Sandra Castillo-Uribe[1], Jorge Salvador-Carlos[1], José Alcocer-Cañez[2], Nelson Cheng[3]

[1] Laboratorio de Biología Molecular y Cáncer, Instituto de Ingeniería, Universidad Autónoma de Baja California, Blvd. Benito Juárez y Calle de la Normal s/n, Mexicali C.P. 21040, Baja California, México

[2] Coordinación Clínica de Cirugía, Hospital General de Zona No. 30, Instituto Mexicano del Seguro Social (IMSS), Av. Lerdo de Tejada s/n, Mexicali C.P. 21100, Baja California, México

[3] Magna International Pte Ltd., 10 H Enterprise Road, Singapore 629834, Singapore

* Correspondence: benval@uabc.edu.mx

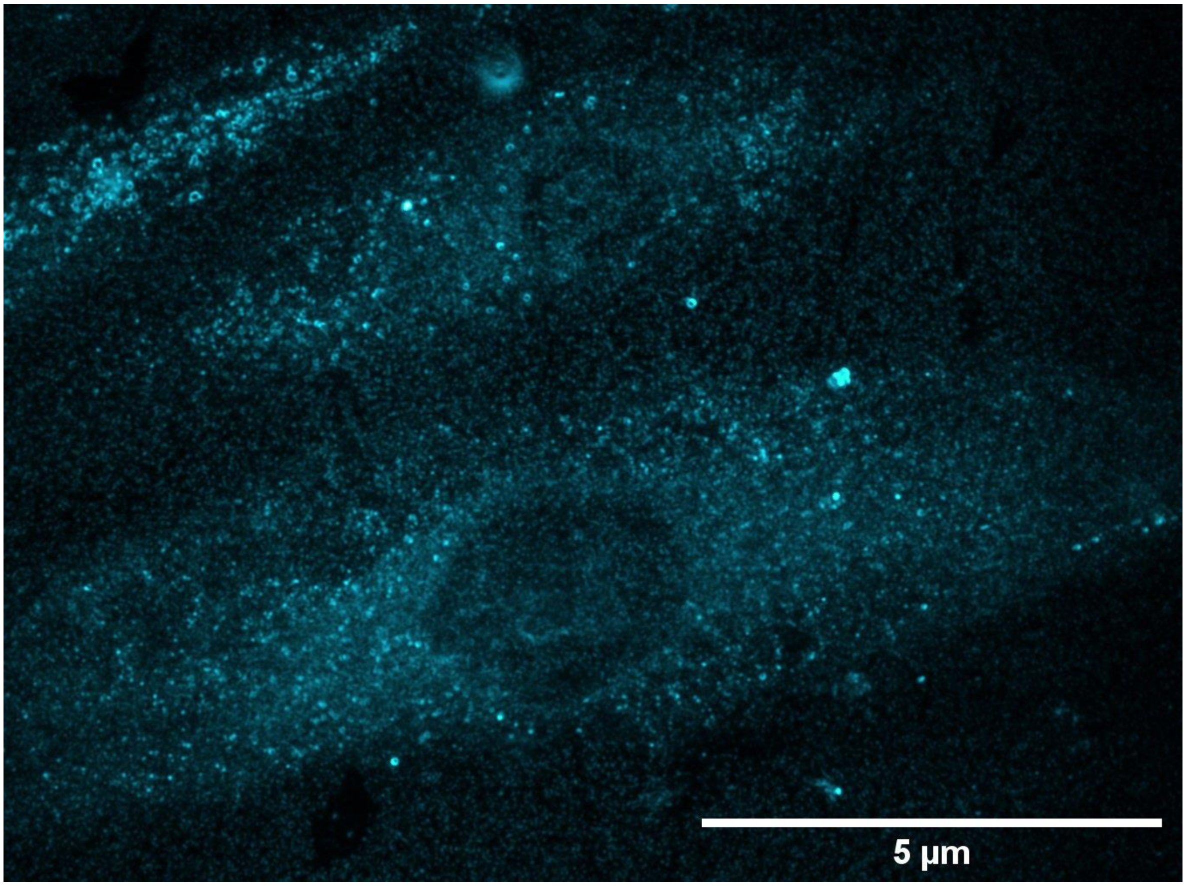

Figure S1. Immunofluorescence micrograph of the endothelial receptor to the von Willebrand factor of the isolated AThEC shows a positive response.
